# Neural Progenitor Cells Expressing Herpes Simplex Virus-Thymidine Kinase for Ablation Have Differential Chemosensitivity to Brivudine and Ganciclovir

**DOI:** 10.3389/fncel.2021.638021

**Published:** 2021-12-06

**Authors:** Zijian Lou, Alexander Post, Christopher E. Rodgers, Mahmood Chamankhah, James Hong, Christopher S. Ahuja, Mohamad Khazaei, Michael G. Fehlings

**Affiliations:** ^1^Division of Genetics and Development, Krembil Research Institute, University Health Network, Toronto, ON, Canada; ^2^Institute of Medical Sciences, University of Toronto, Toronto, ON, Canada; ^3^Department of Surgery, University of Toronto, Toronto, ON, Canada

**Keywords:** spinal cord injury, cell ablation, neural progenitor cell, ganciclovir, induced pluripotent stem cells, brivudine

## Abstract

Neural progenitor cell (NPC) transplants are a promising therapy for treating spinal cord injury (SCI), however, their long-term role after engraftment and the relative contribution to ongoing functional recovery remains a key knowledge gap. Selective human cell ablation techniques, currently being developed to improve the safety of progenitor cell transplant therapies in patients, may also be used as tools to probe the regenerative effects attributable to individual grafted cell populations. The Herpes Simplex Virus Thymidine Kinase (HSV-TK) and ganciclovir (GCV) system has been extensively studied in the context of SCI and broader CNS disease. However, the efficacy of brivudine (BVDU), another HSV-TK prodrug with potentially reduced bystander cytotoxic effects and *in vivo* toxicity, has yet to be investigated for NPC ablation. In this study, we demonstrate successful generation and *in vitro* ablation of HSV-TK-expressing human iPSC-derived NPCs with a >80% reduction in survival over controls. We validated an HSV-TK and GCV/BVDU synergistic system with iPSC-NPCs using an efficient gene-transfer method and *in vivo* ablation in a translationally relevant model of SCI. Our findings demonstrate enhanced ablation efficiency and reduced bystander effects when targeting all rapidly dividing cells with combinatorial GCV and BVDU treatment. However, for use in loss of function studies, BVDU alone is optimal due to reduced nonselective cell ablation.

## Introduction

Neural progenitor cell (NPC) transplants are an exciting therapy for numerous neurodegenerative conditions including traumatic spinal cord injury (SCI; Ahuja et al., 2017a, b). Although there is evidence that NPC transplants lead to improved functional recovery, the roles of the differentiated cells post-engraftment and the mechanisms by which they enhance regeneration of the spinal cord remains a key knowledge gap. Suicide gene systems, also known as gene-directed enzyme prodrug therapies (GDEPT), are currently being developed to eliminate tumors by delivering a gene encoding an exogenous protein into targeted cells. The exogenous protein can then catalyze the conversion of a prodrug into a cytotoxic compound, thus ablating transfected tumor cells. These systems have also been studied for use in transplant therapies, providing a failsafe mechanism for cells in the event that they form teratomas (Jones et al., 2014; Greco et al., 2015; Yagyu et al., 2015; Liang et al., 2018; Kojima et al., 2019). The selectivity and effectiveness of these suicide-gene systems can also be used to elucidate the regenerative effects associated with individual cell populations originating from the graft post-transplantation through loss of function experiments.

### The HSV-TK System

Currently, one of the most widely studied suicide gene systems is the herpes simplex virus-thymidine kinase (HSV-TK) system. The viral thymidine kinase is understood to have approximately 1,000-fold greater affinity for the initial phosphorylation step than endogenous cellular thymidine kinases, preventing general cytotoxicity in non-HSV-TK expressing cells at concentrations of the prodrug that are lethal to cells expressing the viral-derived thymidine kinase (Balzarini et al., 1993; Zhang et al., 2015; Bagó et al., 2016). Due to issues with prodrug pharmacokinetics in the CNS, other thymidine kinase and prodrug combinations have been investigated, including the thymidine kinase of the tomato plant and the prodrug azidothymidine (Stedt et al., 2015). HSV-TK transgenically expressed in transplanted cells interacts with the nucleoside analogue prodrugs ganciclovir, a purine analogue (9-[[2-hydroxy-1-(hydroxymethyl)ethoxy]methyl] guanine; GCV), and brivudine, a pyrimidine analogue [(E)-5-(2-bromovinyl)-2’-deoxyuridine; BVDU; De Clercq, 2005; Dachs et al., 2009; Zhang et al., 2015]. Although HSV-TK interacts with other prodrugs; including acyclovir, penciclovir, and valacyclovir; here we chose to investigate GCV, the most commonly used prodrug for this system, and BVDU, which has been reported to have increased specificity for transfected cells when compared to the others (Denny, 2003).

**Figure 1:**
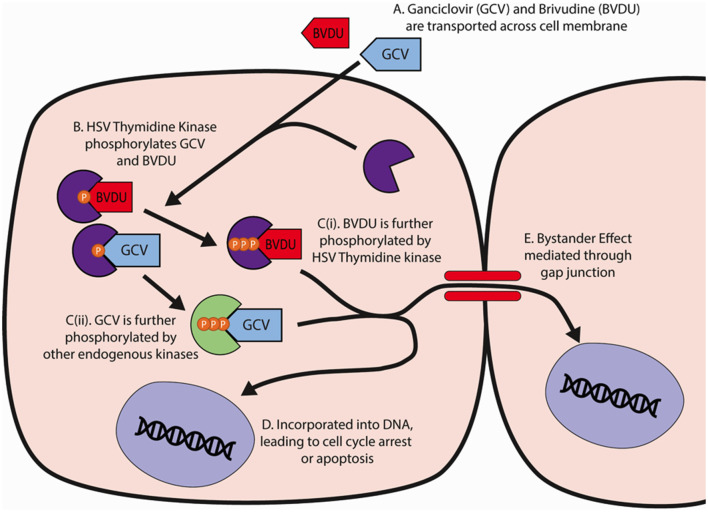
HSV-TK and GCV/BVDU mechanism of action in cell ablation. **(A)** GCV and BVDU travel through the cell membrane into a cell containing the HSV-Thymidine Kinase. **(B)** The viral thymidine kinase will catalyze the initial phosphorylation of GCV and BVDU. **(C)** GCV and BVDU will be further phosphorylated by endogenous kinases and viral kinases respectively, creating nucleoside analogues. **(D)** The triphosphate forms of GCV and BVDU will be incorporated into DNA, and due to their instability, cause DNA backbone breakages that will lead to cell cycle arrest or apoptosis. **(E)** GCV and BVDU may be transported through gap junctions, mediating further cell death in neighboring cells. GCV, ganciclovir; BVDU, brivudine.

GCV and BVDU induce ablation through the actions of their triphosphate forms, which are produced through phosphorylation, first from HSV-TK then from either a continued use of the HSV-TK kinase (BVDU) or endogenous cellular kinases (GCV; De Clercq, 2005; Dachs et al., 2009; Figure 1). Once converted to their triphosphate forms, GCV and BVDU are subsequently incorporated into genomic and mitochondrial DNA strands during cell division and mitochondrial turnover, respectively, and induce chain termination and apoptosis (Balzarini et al., 1993; Beltinger et al., 1999; Fischer et al., 2005; Zhang et al., 2015; Bagó et al., 2016).

A drawback of the HSV-TK^+^GCV/BVDU cell ablation system for neural regeneration studies is the presence of a “bystander effect”. This refers to the additional non-selective ablation of cells neighboring HSV-TK-expressing cells *via* the diffusion of phosphorylated forms of the prodrugs between cells, thought to occur primarily through gap junction intercellular channels (GJICs), particularly connexin 43 (Cx43; Dilber et al., 1997; Mesnil and Yamasaki, 2000; Burrows et al., 2002; van Dillen et al., 2002; Asklund et al., 2003). However, BVDU has been shown to possess a reduced bystander effect compared to GCV due to its reduced incorporation into the DNA of non-transfected neighboring cells (Degrève et al., 1999). The mechanism behind the depressed bystander effect of this pyrimidine analogue is thought to be due to the difference in phosphorylation characteristics between BVDU and GCV. Unlike GCV, which can be phosphorylated from GCV monophosphate to its diphosphate and triphosphate forms by both cellular and viral thymidine kinases, BVDU monophosphate can only be further phosphorylated by the viral thymidine kinase to its diphosphate and subsequent triphosphate form. If only monophosphorylated nucleosides can be transferred through gap junctions to neighboring non-transfected cells, BVDU cannot be incorporated into bystander cell DNA as these cells lack the thymidine kinase activity associated with HSV-TK (Degrève et al., 1999; Dachs et al., 2009).

### Alternative Ablation Systems

Another of the most commonly studied GDEPTs is the Cytosine Deaminase (CD) suicide gene system, which deaminases the prodrug, 5-fluorocytosine, into 5-fluorouracil, and causes death through a similar mechanism to the HSV-TK system. 5-fluorouracil can then diffuse passively into surrounding cells, leading to a greater bystander effect than the HSV-TK system and making it less desirable as a selective cell ablation technique for studying single cell populations (Zhang et al., 2015). The nitroreductase (NTR) suicide gene system has also been studied extensively, and its resulting metabolites are cell membrane permeable as well, leading to a bystander effect that is also too excessive for investigating single cell populations (Bridgewater et al., 1997). Thus, HSV-TK is potentially the best candidate GDEPT for loss of function experiments investigating the role of transplanted cells.

### Selective Cell Ablation

Currently, it remains unclear as to what degree short and long-term mechanisms contribute to physical repair and functional recovery post-SCI. Functional recovery may be a result of NPC integration into host tissues, i.e., the establishment of synaptic connectivity and remyelination by transplanted NPC-derived oligodendrocytes. Alternatively, post-SCI recovery may be driven by the combination of multiple short-term mechanisms such as: (a) trophic support through the secretion of positive growth factors, including BDNF and GDNF; and (b) immune modulation *via* the downregulation of IL-β and TNF-α production. (Siddiqui et al., 2015; Khazaei et al., 2017, 2020). Expressing the HSV-TK suicide-gene under specific cell population promoters, such as Myelin-Basic Proteinpromoters (MBP) for mature oligodendrocytes, can ablate a target cell population after transplantation. By comparing host-graft integration and functional recovery of animals receiving cells to be ablated with controls, the contributions of different mechanisms of repair and regeneration in SCI can be investigated (Curado et al., 2008; Zhang et al., 2015).

The HSV-TK/GCV system has been previously used to ablate rat glioma cells (C6 cell line), murine cerebellar neural stem cells (C17.2 cell line), murine GFAP-expressing definitive neural stem cells, and human-ESC-derived, Ki67^+^ NPCs (Imura et al., 2003; Morshead et al., 2003; Li et al., 2005; Pu et al., 2011; Sachewsky et al., 2014; Tieng et al., 2016). The use of BVDU to ablate neural cells is less common than GCV, having never been used with iPSC-NPCs. BVDU thus far has largely involved the treatment of herpetic encephalitis where infected neurons contain viral HSV-TK expression, as opposed to the transgenic, cellular-driven, GCV system (Wigdahl et al., 1983, 1984; Rosato and Leib, 2015). The HSV-TK^+^GCV ablation system has been used in SCI mice to successfully target endogenous neuron-glial antigen 2^+^ (NG2^+^) pericytes and OPCs, endogenous GFAP^+^ reactive astrocytes, as well as human iPSC-derived NPCs (hiPSC-NPCs; Faulkner et al., 2004; Hesp et al., 2018; Kojima et al., 2019). Therefore, we conducted a preliminary proof-of-concept study to evaluate and compare the targeted cell-killing effects of GCV and BVDU against hiPSC-NPCs *in vitro* and *in vivo*, providing a key methodological advancement in optimizing HSV-TK mediated ablation of translationally relevant human iPSC-derived NPCs in a neural regeneration context.

## Materials and Methods

### Generation of Stable HSV-TK Expressing NPCs

The following HSV-TK system plasmids were obtained from Addgene: (1) The Tol2 transposon plasmid, pKTol2P-PTK (Addgene #85599), containing a gene for a fusion protein of HSV1-TK and puromycin resistance under the control of the ubiquitous PGK promoter; and (2) the Tol2 transposase containing plasmid, pCMV-Tol2 (Addgene #31823; Chen and Bradley, 2000; Balciunas et al., 2006; Clark et al., 2007), required for Tol2-based genetic integration.

HSV-TK transfection of human iPSC-derived NPCs (derived from hiPSC line BC1 using the dual SMAD inhibition method) was accomplished through electroporation using the Amaxa Nucleofector 2 (Lonza). 1.0 × 10^6^ NPCs were loaded into a curved glass electroporation cuvette with 1 μg (5 μl) of both pCMV-Tol2 and pKTol2P-PTK plasmid DNA, and 100 μl of Nucleofector® Solution. Program A33 on the electroporator was selected, and after completion of the transfection, 500 μl of pre-warmed, 37°C B27N2 media was added to each cuvette. Cells were then purified by the addition of puromycin (Gibco^TM^ Puromycin Dihydrochloride) to the B27N2 media at 10 μg/ml at 4 days post-transfection.

To make stable cell lines expressing HSV-TK, a Tol2 transposon-based gene transfer vector was used (Kawakami et al., 2016). A transposon-based gene transfer vector for genomic integration into hiPSC-derived NPCs is considered a safe and reliable method as compared to lentiviral-based vectors (Di Matteo et al., 2012; Vargas et al., 2016). This system consisted of pCMV-Tol2—containing the Tol2 transposase, and pkTol2P-PTK—containing the Tol2 transposon carrying a puromycin-resistance-HSV-TK fusion protein gene. Upon generation of stable hiPSC-NPCs, monoclonal cell lines were derived by culturing cells in clonal density (10 cells/ml) for three passages (Coles-Takabe et al., 2008). Purified, monoclonal lines were expanded, and expression of HSV-TK was confirmed by the RFP reporter.

### HSV-TK-NPC GCV and BVDU Ablation Assay

For both GCV and BVDU, 5.0×10^4^ HSV-TK^+^ NPCs were plated in 2 ml of B27N2 media in each well of four 6-well culture plates pre-coated with Matrigel. After 24 h, the media of all plates was changed, and the wells of each plate received the following six concentrations of GCV or BVDU dissolved in B27N2 media, in one well each: 0 μg/ml (negative control well); 0.0625 μg/ml; 0.125 μg/ml; 0.25 μg/ml; 0.50 μg/ml; and 1.0 μg/ml.

Once daily for a period of 96 h, one plate of HSV-TK^+^ NPCs for both prodrug treatments (two plates per prodrug/day) were removed from 37°C incubation and the cells were fixed with 2 ml of 4.0% PFA (Sigma-Aldrich), washed, and left in 1× PBS. All wells were then stained with 1–2 μg/ml of DAPI for 30 min as a nuclear counterstain. Wells were imaged with the brightfield channel of an EVOS FLoid^TM^ Cell Imaging Station before fixation, and with the brightfield and blue fluorescent channels post-fixation. A minimum of five representative images was acquired per well. Estimation of the total remaining attached cells/well was accomplished through “peak” analysis, whereby DAPI-stained nuclei (“peaks”) were counted in ImageJ photo-analysis software. The mean number of total attached cells/well was determined by extrapolating the mean number of cells in the surface area (SA) covered by a representative image (approximately 0.37 mm^2^) to the total SA per well (approximately 1134 mm^2^). The same procedure was repeated for the 48, 72, and 96-h timepoints, and *n* = 3 replicates were performed for the entire experiment. All concentrations were normalized to the 0 drug concentration control for each time point. Statistical analyses (one-way and two-way ANOVAs with Bonferroni *post hoc* corrections) were performed using GraphPad Prism version 6 software.

### HSV-TK-NPC GCV and BVDU Bystander Ablations

A mixed culture of HSV-TK^+^ NPCs and wild-type (WT) GFP^+^-NPCs in a 1:1 ratio was made, wherein a total of 5.0 × 10^4^ cells were plated in each well (pre-coated with Matrigel) of two 6-well culture plates. After 24 h, each plate received the following six concentrations of GCV or BVDU into one well each, with daily replacement: 0 μg/ml (negative control well); 0.0625 μg/ml; 0.125 μg/ml; 0.25 μg/ml; 0.50 μg/ml; and 1.0 μg/ml. All wells were imaged once every 24 h with brightfield and green fluorescent channels of an EVOS FLoid^TM^ Cell Imaging Station until the 96-h end point.

### Bystander Effect TUNEL Assay

To further investigate the extent of bystander-effect mediated cell death associated with GCV, BVDU, and a combinatorial drug treatment, 1:1 HSV/TK^+^ and wild-type NPCs expressing mCherry were plated on Matrigel-coated 6-well plates at GCV (1 μg/ml), BVDU (1 μg/ml), or a combination of both GCV (0.5 μg/ml) and BVDU (0.5 μg/ml) for 96 h. Cells were then fixed with 4% PFA and apoptotic cells were labeled using a TUNEL Assay Kit (Abcam). Apoptotic cells were obtained by gating for TUNEL positive cells using FACS and the number of apoptotic wild type (mCherry positive) or HSV/TK^+^ cells was quantified.

### Spinal Cord Injury and Cellular Transplants

Adult female RNU rats (200–250 g; *n* = 15) were anesthetized with isoflurane (2–3%) delivered in a 1:1 ratio of oxygen and nitrogen, and rats underwent a C6-C7 laminectomy exposing the spinal cord, after which the dura mater was dissected to allow for easier transplantation. Rats were then transplanted with the HSV-TK transfected human iPSC NPCs prepared as described earlier in the article. Cells were lifted from culture dishes, centrifuged and diluted to a volume of 50,000 cells/μl, and kept on ice for transplant. Cells were injected at a depth of 1 mm into four sites, forming a 2 mm by 2 mm square around a defined epicenter under the C6 vertebrae. Cells were injected at 50,000 cells/μl for 2 μl per site. Muscles and skin were closed with sutures, and animals were treated post-operatively with buprenorphine twice daily for 3 days, meloxicam for 5 days, and 15 ml of saline for 7 days. Twelve days post-transplant, randomly generated groups of animals received tail vein injections of 10 mg/kg of BVDU (*n* = 3), 10 mg/kg GCV (*n* = 3), or 5 mg/kg of BVDU and GCV (*n* = 3) each day for four consecutive days.

### Post-Mortem Tissue Preparation and qPCR

Animals were sacrificed 16 days post-transplantation and spinal cords were harvested. Animals were anesthetized with isoflurane (5%) and transcardially perfused with ice-cold Phosphate Buffered Saline (pH 7.4). Spinal cord sections 6 mm rostral and caudal of injection sites were harvested, dura was removed, and tissue was cut into small pieces, placed in an autoclaved 1.5 ml microfuge tube, snap-frozen in liquid nitrogen, and stored at −80°C. The tissue was then thawed and digested in 500 μl of extraction buffer (Qiagen Blood and Tissue DNA extraction kit) and 50 μl of Proteinase-K solution (10 mg/ml) at 55°C for 2 h with occasional vigorous mixing. The tissue was then homogenized using a sterile blue automated pestle until the solution was fluid with no chunks of undigested tissue. Genomic DNA was then extracted using a Qiagen kit, and pelleted DNA was resuspended in DNase and RNase free sterile water. Alu element presence was then detected and quantified using qPCR.

### Immunohistochemistry

Animals grafted with HSV-TK expressing NPCs were sacrificed 16 days post-transplant, with the ablation group being treated with GCV+BVDU for 4 days before sacrifice. Animals were perfused with 4% PFA and the spinal cords were harvested and cryosectioned. Tissue sections were blocked with 5% skim milk powder, 1% BSA, and 0.3% Triton X-100 for 1 h before being incubated with the following primary antibodies at 4°C overnight: Anti-human nuclear antigen [235-1] (ab191181; 1:500) and Anti-Ki67 (ab15580; 1:500). Sections were then washed with 1× PBS before being incubated in the fluorescein Roche *in situ* Cell Death Detection Kit enzyme-labeling solution prepared according to manufacturer’s instructions for 1 h at 37 degrees in the dark. Following further 1× PBS washes, the sections were labeled with Alexa secondary antibodies and DAPI (1:300) for 1 h at room temperature in the blocking solution. Coverslips were mounted onto the glass slides with Mowiol and sections were images using a confocal microscope.

### Statistics

All quantitative data are expressed as mean ± SEM. All experiments were performed using a minimum of three biological and three technical replicates. Differences between groups were assessed by one- and two-way analysis of variance (ANOVA) with Bonferroni *post hoc* test to correct for multiple comparisons (*p* < 0.05). Data were analyzed with GraphPad Prism (GraphPad Software Inc., La Jolla, CA, USA,^1^), R x64 3.5.3^2^ and FlowJo Software (Becton, Dickinson and Company, Life Sciences, Ashland, OR, USA^3^).

## Results

### GCV and BVDU Successfully Ablate HSV-TK^+^ Human iPSC-Derived NPCs

**Figure 2:**
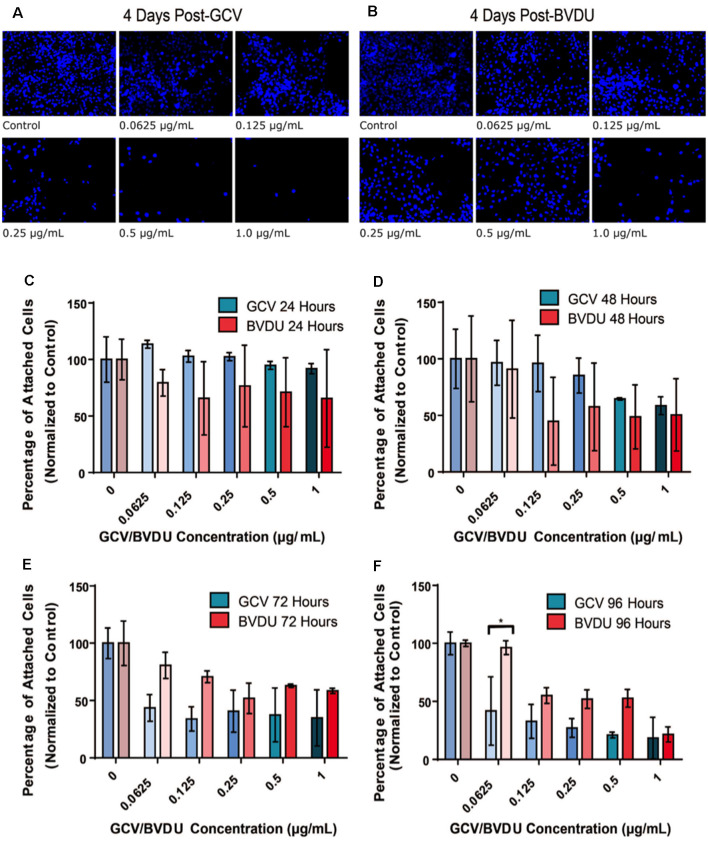
Number of attached HSV-TK NPCs following different durations of GCV treatment. 5.0 × 10^4^ HSV-TK NPCs were plated on each well of two separate, 6-well culture plates and subjected to increasing concentrations of BVDU or GCV (0.0625–1 μg/ml) through media changes after 24 h. Panels **(A)** and **(B)** represent the 96-h time point, wherein DAPI^+^ cells are stained and concentrations of GCV and BVDU are indicated in the bottom left of the image. Cell death can be seen in all conditions except for the control. Panels **(C–F)** display the quantification of cell count data from ablation experiments. Panels show a direct comparison between GCV (turquoise color scheme) and BVDU (red color scheme) at the four collected time points 24 h **(C)**, 48 h **(D)**, 72 h **(E)**, and 96 h **(F)**. Data were normalized to the control of each timepoint (0 μg/ml GCV or BVDU), where the control represents 100 percent cell survival and treatment conditions are represented as a percent of the control. To statistically analyze the data, we performed Two-way ANOVAs with Bonferroni *post hoc* corrections. *P < 0.05. NPC, neural progenitor cell.

First, we evaluated the cell-killing efficiency of both the GCV and BVDU prodrugs on HSV-TK^+^ NPCs, *via* DAPI staining of the total attached cells remaining after various timepoints and concentrations of GCV/BVDU treatment (Figure 2 and Supplementary Figure 1). As shown in Figure 2 and Supplementary Figure 1, 96-h treatments of the HSV-TK transformed hiPSC-NPCs with 1 μg/ml of either GCV or BVDU reduced the number of attached cells remaining per well by over 80% when compared to control wells (Figures 2A,B; Supplementary Figures 1A,B).

Quantification of the ablation efficiency of both GCV and BVDU was set up as described in the “HSV-TK-NPC GCV and BVDU Ablation Assay” section and displayed in Figure 2 and Supplementary Figure 1. The remaining numbers of HSV-TK NPCs in each well were normalized to the 0 drug control of that time point to account for cell proliferation after the addition of either drug. After 96 h of GCV treatment, all concentrations used were found to significantly reduce the mean number of attached cells remaining compared to the control well (Supplementary Figure 1B; *p* < 0.05). BVDU ablation did not show similar results (Supplementary Figure 1A), although the three highest concentrations used (0.25 μg/ml, 0.5 μg/ml, and 1 μg/ml) showed a trend towards reducing the number of attached cells remaining, from the 48 h time point onwards. When comparing drug effectiveness across concentrations and time points (Figures 2C–F), no significant differences in the quantities of attached cells were detected except with a drug concentration of 0.0625 μg/ml at the final time point. Although statistical significance was not reached, a trend can be seen beginning at the 72-h time point and through to the end of the experiment where GCV displays a higher killing efficiency compared to that of BVDU (Figures 2C–F). This was later confirmed using Fluorescence Activated Cell Sorting (FACS; Figure 4).

**Figure 3:**
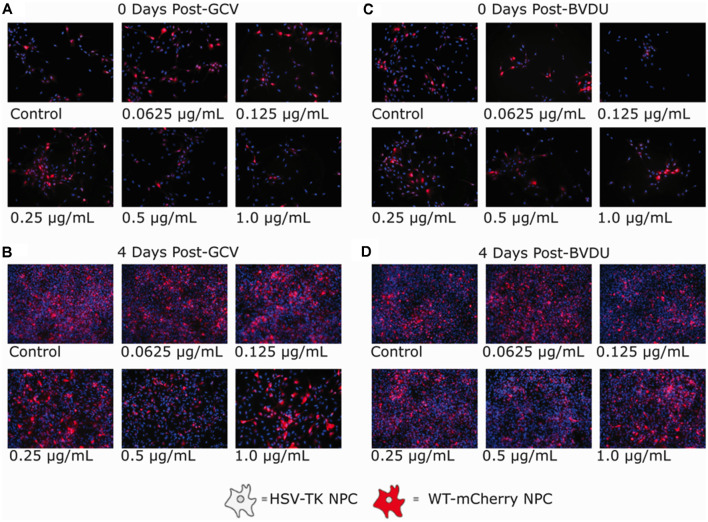
Ganciclovir and brivudine bystander effect assays of HSV-TK^+^ NPCs and WT-mCherry^+^ NPCs. 5.0 × 10^4^ cells of a 1:1 mixture of HSV-TK^+^ and WT-mCherry^+^ NPCs were plated in each well of a 6-well plate. Twenty-four hours after plating, cells were treated with 0.0625, 0.125, 0.25, 0.5, and 1.0 μg/ml of either Ganciclovir or Brivudine **(B)** and **(D)** and a negative control **(A)** and **(C)**. Ninty-six hours post GCV or BVDU treatment, non-fluorescent spots of DAPI stain are not visible indicating HSV-TK cell death. The mCherry expression is shown in red.

**Figure 4:**
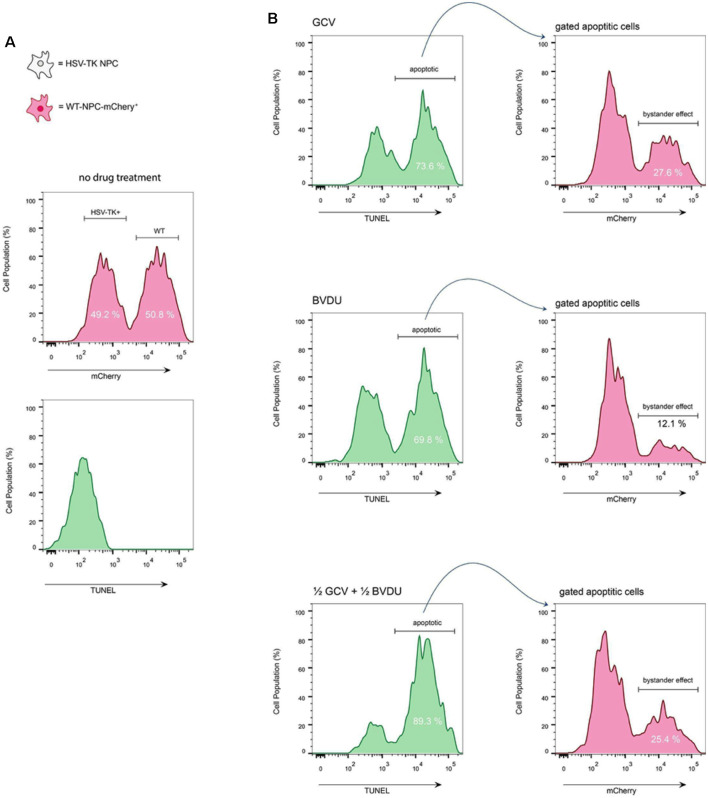
TUNEL assay of co-cultured HSV-TK^+^ NPCs and WT-mCherry^+^ NPCs. **(A)** To assess the bystander effect by either GCV or BVDU, wild type NPCs that express mCherry were co-cultured with HSV-TK expressing NPCs at a 1:1 ratio. Analysis of the cells before treatment with drugs shows the populations of both WT and HSV-TK cells to be close to 50% (top). Staining untreated cells with a TUNEL kit shows no apoptotic cells at this stage (bottom). **(B)** A mixed culture of cells was treated with the highest effective dose of either GCV (1 μg/ml; top panel), BVDU (1 μg/ml; middle panel), or a combination of both at a 1:1 ratio (0.5 μg/ml GCV and 0.5 μg/ml of BVDU; bottom panel). After 96 h, all cells, including both attached and floating, were fixed with 4% PFA, and stained with TUNEL kit.

### GCV and BVDU Exert a Bystander Effect on HSV-TK^+^ Human iPSC-Derived NPCs

Next, we analyzed the presence of a bystander cell-killing effect for either GCV or BVDU using a mixed cell culture of HSV-TK-transformed NPCs and wild-type NPCs fluorescently tagged with mCherry. The selectivity of the HSV-TK^+^GCV/BVDU ablation system for HSV-TK-transformed NPCs would have an impact on designing future *in vivo* experiments.

GCV (Figures 3A,B) and BVDU (Figures 3C,D) treatment of the mixed NPC populations for 96 h led to a noticeable decrease in the number of mCherry^+^ attached NPCs (increased presence of DAPI stain without mCherry fluorescence). Although a bystander effect was apparent for both prodrugs in combination with HSV-TK-transformed NPCs in this assay, the rapid proliferation rates of both the HSV-TK^+^NPCs and mCherry^+^ WT-NPCs (Supplementary Figure 2) in combination outpaced cell death. For GCV treated wells, the bystander effect increased in intensity as the concentration of drug increased, until 0.5 μg/ml where it leveled off. BVDU followed a similar trend although the intensity of the bystander effect was observed to be slightly less, particularly at the higher concentrations of 0.5 μg/ml and 1 μg/ml. However, when performing the same experiment using a TUNEL assay, we found that there were differences in bystander effect and killing efficiency between GCV and BVDU at 1 μg/ml. GCV was found to ablate cells approximately 4% more than BVDU, and the bystander killing effect of GCV was 27.6% compared to 12.1% with BVDU. We observed the highest killing efficiency of 89.3% when using a combination of 0.5 μg/ml of GCV and 0.5 μg/ml of BVDU, while the bystander effect was observed to be 25.4%, which was 2.2% below that of GCV alone (Figure 4B).

### GCV and BVDU Ablate HSV-TK^+^ Human iPSC-Derived NPCs *In vivo*

**Figure 5:**
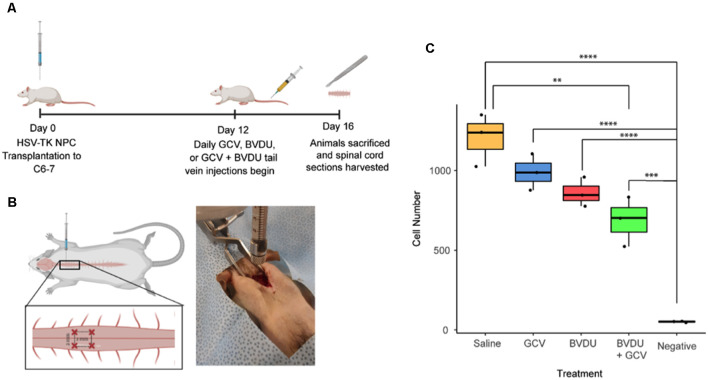
Ablation efficiency of GCV and BVDU on transplanted HSV-TK^+^ NPCs in a rat model. **(A)** HSV-TK expressing NPCs were transplanted into a rat model of spinal cord injury, and tail vein injections of 10 mg/kg of BVDU (*n* = 3), 10 mg/kg of GCV (*n* = 3), or 5 mg/kg of both BVDU and GCV (*n* = 3) were given every day for 4 days at 12-days post-transplant. **(B)** Rats received four injections of NPC transplants at the C6–7 laminae of the cord, two injections on each side with ipsilateral injections being 2 mm apart from each other and 1 mm away from the center of the spinal cord. **(C)** Following the conclusion of injections, at 16 days post-transplant, cell survival was assessed by quantifying Alu element copy number from gDNA extracted from harvested spinal cord sections. Rats receiving combinatorial GCV and BVDU injections displayed a significant reduction in overall cell number as compared to the saline control (*P* < 0.01). Individual administration of GCV or BVDU did not lead to a significant decrease in cell number. Data were standardized to gDNA concentration per 1 million rat cells. Diagrams **(A)** and **(B)** were created with biorender.com. **P < 0.01, ***P < 0.001, ****P < 0.0001.

To assess the ablation efficiency of the HVS-TK system *in vivo*, we transplanted HSV-TK NPCs into the RNU rat model to test ablation efficiency. Cells were prepared and injected into the C6 level of the spinal cord, and 12 days later, given tail vein injections of 10 mg/kg of BVDU (*n* = 3), 10 mg/kg of GCV (*n* = 3), or 5 mg/kg of both BVDU and GCV (*n* = 3) each day for four consecutive days. Rats were sacrificed 16 days post-transplant, a 12 mm section of cord centered on the grafts was harvested, and Alu element quantification was performed by qPCR to determine the number of surviving HSV-TK^+^ hiPSC-NPCs. All treatments led to a decrease in the number of cells remaining, with the combinatorial injections of both drugs reaching statistical significance when compared to the saline control (*P* < 0.01; Figure 5).

### GCV and BVDU Combinatorial Treatment Reduces Ki-67 Staining in HSV-TK Expressing NPCs *In vivo*

To visualize the proportion of actively proliferating HSV-TK expressing NPCs and cells that are undergoing apoptosis, HSV-TK expressing NPCs were transplanted into RNU rats. The animals in the ablation group were treated with a combination of GCV and BVDU for 4 days before all animals were sacrificed 16 days post-transplantation. Ki-67 and a TUNEL kit were used to stain spinal cord sections for actively proliferating cells and cells undergoing apoptosis, respectively. Grafted cells were identified through HNA staining. Treatment with GCV+BVDU led to a large decrease in actively proliferating cells within the graft, as seen with reduced Ki-67^+^ and HNA^+^ cells. Furthermore, the prodrug treated group showed higher TUNEL staining in the tissue which was not specific to the grafted cells.

## Discussion

In this study, we demonstrated the effective elimination of proliferating hiPSC-NPCs using the HSV-TK^+^GCV/BVDU ablation system. We detected an approximately 80% reduction in the estimated number of remaining attached cells when compared to controls for both GCV and BVDU treatments at the highest concentrations used (1 μg/ml) after 96 h *in vitro* (Figure 2F and Supplementary Figure 1). No significant differences were detected between equivalent concentrations of the GCV and BVDU prodrugs after 96 h of treatment in terms of the estimated quantity of attached HSV-TK^+^ NPCs per well of each plate, except in the 0.0625 μg/ml concentration group (Figure 2F). However, our cell ablation assay showed a trend of GCV ablating cells more efficiently than BVDU. This was then confirmed using a TUNEL assay (Figure 4), wherein GCV was found to ablate cells at a rate approximately 4% higher than BVDU. It is important to note that for the remaining attached cells following prodrug treatment during the cell ablation assay, many cells may have been in the process of, or recently underwent, apoptosis and had simply not yet detached, skewing data to represent a higher cell survival than what would be seen at a molecular level (Figures 2A,B). Lastly, we observed that GCV and BVDU combinatorial treatment ablates transplanted HSV-TK NPCs in the spinal cord of rats.

This study builds on previous work by Kojima et al. to report on the usage of the HSV-TK^+^GCV system for ablating hiPSC-NPCs. Previously, the HSV-TK^+^GCV enzyme-prodrug ablation system has been utilized to target such neural cell types as: C17.2 immortalized mouse cerebellar NPCs, C6 rat gliomas, GFAP-expressing mouse dendritic NSCs, endogenous murine NG2^+^ pericytes and OPCs, murine GFAP^+^ reactive astrocytes, human ESC-derived NPCs, and hiPSC-NPCs (Morshead et al., 2003; Faulkner et al., 2004; Li et al., 2005; Uhl et al., 2005; Pu et al., 2011; Tieng et al., 2016; Hesp et al., 2018; Kojima et al., 2019). BVDU-mediated ablation of hiPSC-NPCs is novel as, outside the treatment of herpetic encephalitis for mouse and human neurons, there have not been any reported uses of HSV-TK^+^BVDU ablation for human neural cell types, including NPCs (Wigdahl et al., 1983, 1984; Field et al., 1984). This represents an important area of study, as BVDU-based ablation shows potential advantages over GCV strategies due to reduced toxicity-related bystander effects that could potentially affect *in vivo* ablation studies (De Clercq, 2005).

Our results verify the existence of a bystander effect for both GCV and BVDU prodrugs when utilized to ablate hiPSC-derived NPCs modified to express HSV-TK (Figures 3, 4). These findings are expected, as both prodrugs are known to possess some degree of bystander effect due to GCV-monophosphate and BVDU-diphosphate “spillover” to neighboring cells through Cx43 channels (Dachs et al., 2009). Although BVDU was predicted to have a reduced bystander effect over GCV, we were unable to detect a significant difference between the two systems in the cell ablation assay. However, when confirming these results using a TUNEL assay, our data showed an approximately 15% reduction in the bystander effect of BVDU treated plates compared to GCV. Although the mechanisms of apoptosis induced by GCV and BVDU are understood to be similar, as both are nucleotide analogues inducing apoptosis primarily through incorporation into replicating genomic or mitochondrial DNA, there are minor differences between these mechanisms. For example, BVDU has the ability to target thymidylate synthase while GCV can induce extrinsic Fas-pathway apoptosis [involving the formation of the death-inducing signaling complex (DISC)] without the activation of Fas ligand (Balzarini et al., 1987; Beltinger et al., 1999). These aforementioned differences in the apoptotic pathways induced by GCV and BVDU could be responsible for the differences in the bystander effect and explain why using both drugs in conjunction provides a synergistic effect.

Depending on the goal of the system in question, the bystander effect may be advantageous. The HSV-TK system is commonly studied with the goal of eliminating CNS tumors by taking advantage of the bystander effect by which a small pocket of transfected cells can eliminate surrounding rapidly dividing tissue (Zhang et al., 2015). However, the bystander effect will make it difficult to study the regenerative role of a single cell population originating from a graft in a knockout type of study. After comparing the effects of administering GCV, BVDU, and a combination of both drugs on iPSC-NPCs, we found that we can modify the degree of bystander effect and target cell ablation to fit the needs of the study. Using both drugs in conjunction, we found the highest killing efficiency to be 89.3%, about 16% and 20% higher than GCV and BVDU, respectively. Coupling this with a 25% bystander effect, a combinatorial approach could prove advantageous for eliminating rapidly dividing cancerous tissue (Figures 4A,B). However, if the goal is to study a single population of cells with minimal bystander effects on neighboring cells, the inducible Caspase 9 (iCASP9) suicide gene system may be a viable option. Since the iCASP9 system activates the endogenous apoptosis pathway, it has several advantages over the HSV-TK system. Firstly, it does not require cells to be actively dividing in order to function. Moreover, the iCASP9 system does not directly cause the death of non-transfected neighboring cells through gap junction-mediated transport of the activated drug. Furthermore, the iCASP9 system has reported an *in vitro* ablation rate of 94% to 99%, which is greater than the highest ablation rate we observed in the HSV-TK/GCV+BVDU combinatorial treatment (Yagyu et al., 2015). Unfortunately, the downside of using the iCASP9 system is the very high cost of the dimerization molecule required for inducing the apoptotic pathway in studies with high numbers or using animals with larger masses. Furthermore, the extent to which the dimerization agent penetrates the Blood- Brain Barrier has not been reported, so the system may potentially fail to activate if the cells are in the CNS.

**Figure 6:**
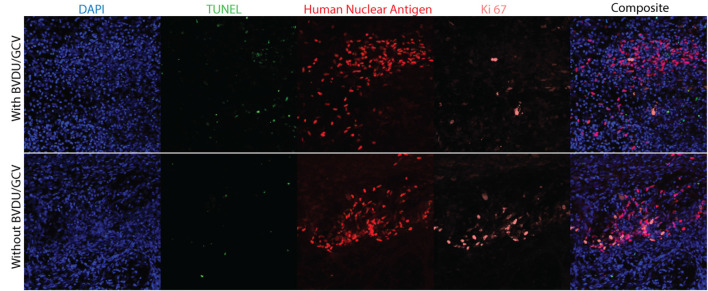
Immunohistology of cell grafts with and without prodrug treatment. Rat spinal cords were collected 16 days post-transplantation, with the ablation animal group receiving 4 days of GCV and BVDU treatment before being sacrificed (*n* = 3). To visualize cell nuclei, apoptotic cells, transplanted NPCs, and actively proliferating cells, the spinal cord sections were stained with DAPI, a fluorescein TUNEL kit, human nuclear antigen, and Ki-67 respectively.

Following transplantation of HSV-TK hiPSC-NPCs into the cervical spine of the rat model, we were able to demonstrate *in vivo* elimination of the cells through administering GCV, BVDU, or a combination of the two drugs (Figure 5). Our results indicate that the largest elimination of the cells occurred when the rats were injected IV with a combination of GCV and BVDU, which reflects the trend that we saw in the *in vitro* TUNEL assay (Figure 4). Even though the combinatorial administration of both drugs resulted in a significant increase in cell elimination, it should be noted that the dosage of the drug that was used was significantly lower than that of most *in vivo* studies on HSV-TK (Williams et al., 2015). The 10 mg/kg per day dose of prodrug was chosen to elucidate the degree of elimination that a combinatorial treatment would have on transplanted HSV-TK^+^ NPCs *in vivo*. However, due to body surface area differences, rats require a dose approximately 6.2 times larger than humans to experience the same effect (Nair and Jacob, 2016). It was observed through immunohistology that the majority of Ki-67^+^ grafted cells were eliminated after 4 days of combinatorial prodrug treatment with minimal TUNEL staining within the graft at 16-day post-transplant time point (Figure 6). TUNEL staining was observed in the endogenous tissue adjacent to the grafted cells as well, which can be attributed to the bystander effect. Thus, to reduce the bystander effect, the prodrug treatment length for ablation can be reduced as almost all proliferating grafted cells were ablated by 4 days of prodrug tail-vein injections. Furthermore, in future studies, the HSV-TK gene may be placed under a promoter that is not active when the cell isn’t proliferating for more controlled expression of the ablation system and further reduced bystander effect (Liang et al., 2018). As BVDU has a lower bystander effect than the combinatorial treatment and GCV treatment alone (Figure 4), it may be employed by itself to reduce endogenous cell death. However, our data indicate that BVDU by itself results in lower cell death than a combinatorial approach, so longer treatment periods should be tested to achieve total ablation of proliferating grafted cells (Figure 5).

A possible explanation for why the two prodrugs have an additive effect when introduced to the cells together is that they are analogues of different nucleoside bases; ganciclovir is an analogue for guanine and brivudine is an analogue of thymidine (Keam et al., 2004; Al-Badr and Ajarim, 2018). When introduced together, their triphosphate forms compete for integration into the host DNA during replication with the host nucleotides but not with each other, increasing the frequency of chain termination and double stranded DNA breaks due to more targets being available.

There is concern that the HSV-TK^+^ NPCs could be expressing the fail-safe system unevenly due to the random integration of the transgene into the NPC DNA. As with all random integration with ubiquitous promoters, transgene silencing is an issue that results in subpopulations of the cell line expressing different levels of the HSV-TK protein. Thus, although this cell line is acceptable for use in validating ablation efficiency of different prodrug options, future work is needed to produce a cell line with sufficient expression of the fail-safe system in every cell to ensure optimal ablation when the prodrug is introduced.

Ultimately, the HSV-TK^+^ system that is being optimized for the hiPSC-NPCs in our study could see potential use for *in vivo* ablations of cells transplanted in rodent models of SCI. Through ablation of transplanted HSV-TK^+^ NPCs in SCI-model rats, questions relating to NPC transplantation for SCI neuroregeneration may be answered. Specifically, the issues of: (1) short-term trophic support and immunomodulation vs. long term integration; (2) tumor generation from iPSC-NPC transplants and treatment mechanisms; and (3) the amount and timing of proliferation required for transplanted hiPSC-NPCs to yield sufficient quantities of trophic/immune-modulating factors and NPC-derived cells necessary to provide effective neuroregeneration post-SCI. Such cell type-specific ablation could elucidate the contributions of different mechanisms of regeneration and repair to observed improvements in neurobehavioral function following NPC transplantation into the injured spinal cord.

## Conclusion

In summary, we present a proof-of-concept demonstration of the HSV-TK^+^GCV/BVDU ablation system with hiPSC-NPCs, and an efficient ablation assay. We anticipate that this work will have important implications when studying methods to control oncogenic tissue proliferation and when assessing the role of proliferating hiPSC-NPCs in mediating regenerative effects post-transplantation.

## Data Availability Statement

The raw data supporting the conclusions of this article will be made available by the authors, without undue reservation.

## Ethics Statement

The animal study was reviewed and approved by the University Health Network Research and Ethics Board.

## Author Contributions

ZL, AP, CR, MC, and CSA conducted experiments. ZL, AP, and JH analyzed the data and performed statistics. CR wrote the original draft. CSA, ZL, CR, and AP reviewed and edited the draft. MK and MGF conceptualized this study and established methodology and supervised this study. MGF acquired funding. All authors contributed to the article and approved the submitted version.

## Conflict of Interest

The authors declare that the research was conducted in the absence of any commercial or financial relationships that could be construed as a potential conflict of interest.

## Publisher’s Note

All claims expressed in this article are solely those of the authors and do not necessarily represent those of their affiliated organizations, or those of the publisher, the editors and the reviewers. Any product that may be evaluated in this article, or claim that may be made by its manufacturer, is not guaranteed or endorsed by the publisher.

## Footnotes

^1^ http://www.graphpad.com

^2^ https://cran.r-project.org/bin/windows/base/old/3.5.3/

^3^ https://www.flowjo.com/
