# Supplementary material for: Neural Progenitor Cells Expressing Herpes Simplex Virus-Thymidine Kinase for Ablation Have Differential Chemosensitivity to Brivudine and Ganciclovir
Source: Front Cell Neurosci. 2021 Dec 6;15:638021. doi: 10.3389/fncel.2021.638021 (PMC8685296; doi:10.3389/fncel.2021.638021)
Supplement: Supplementary file 1 [file Data_Sheet_1.PDF]

Supplemental Figures

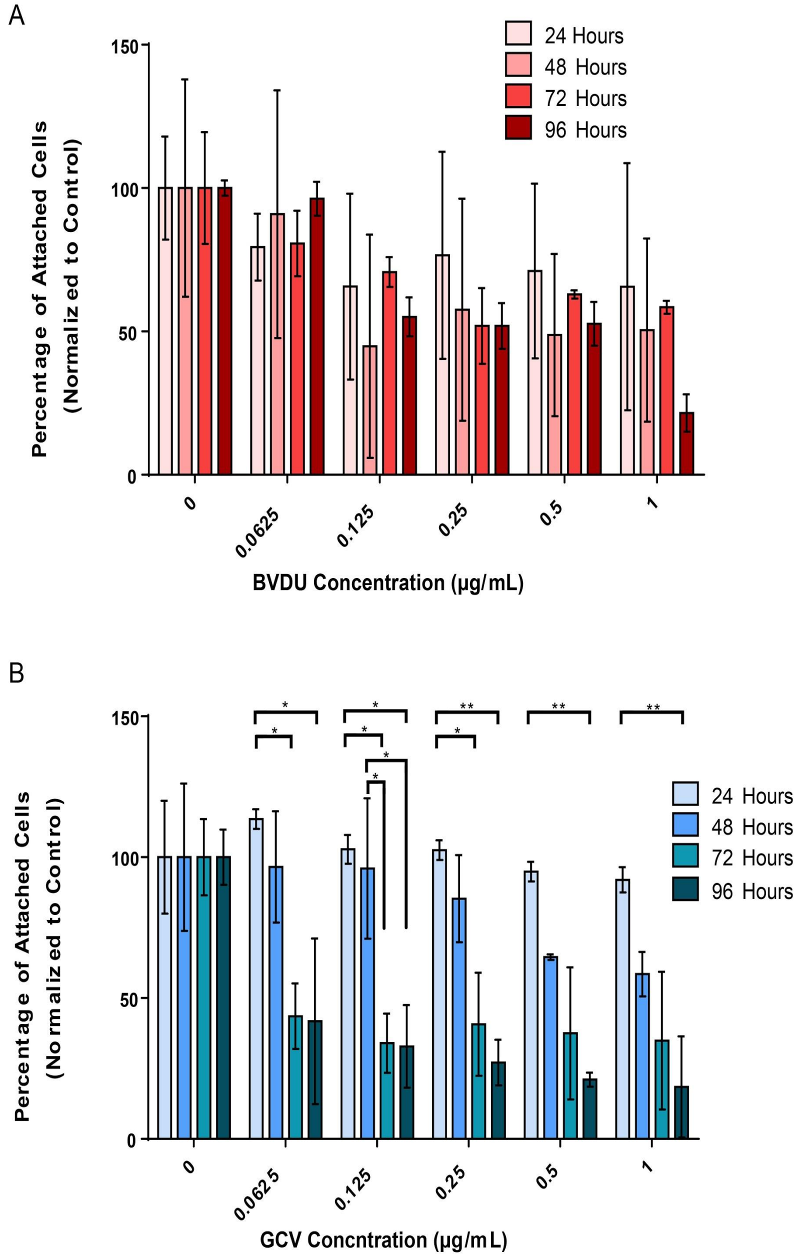

Supplemental Figure 1. Quantification of GCV and BVDU Ablation efficiency on iPSC-NPCs. Panels **A** and **B** display the quantification of cell count data from BVDU (**A**) and GCV (**B**) ablation experiments. Data was normalized to the control of each timepoint (0 µg/mL GCV or BVDU), where the control represents 100 percent and treatment conditions are represented as a percent of the control. To statistically analyze the data we performed One-way ANOVAs with Bonferroni post-hoc corrections. $P<0.05$.

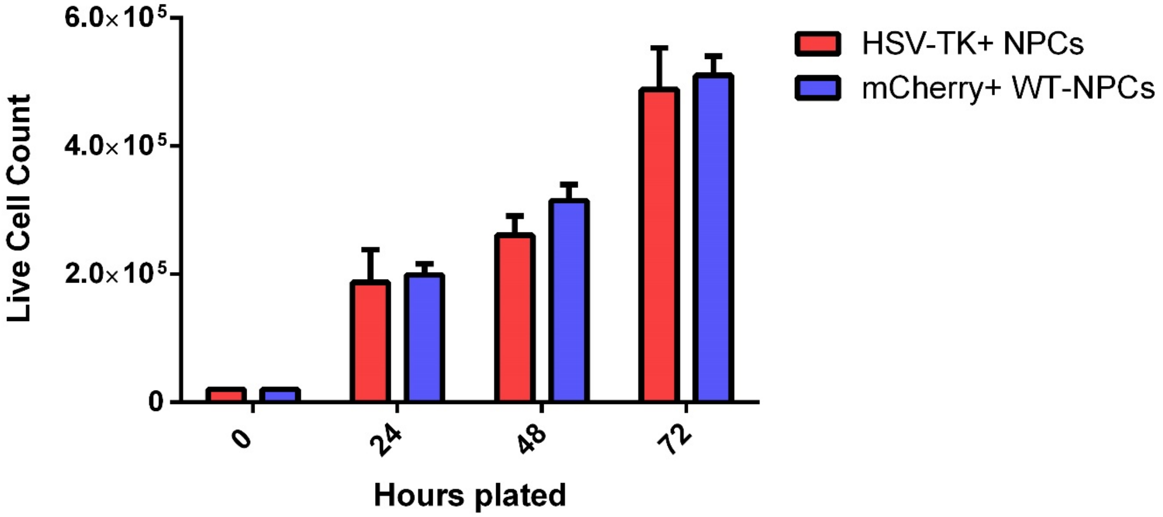

Supplemental Figure 2. Quantification of HSV-TK+ iPSC-NPCs and mCherry+ WT NPCs Proliferation Rates. 50 000 HSV-TK+ or mCherry+ NPCs were plated in 12 Matrigel-coated wells. Cells were then grown for 96 hours and every 24 hours 3 wells of each cell type were lifted and counted using a TC20 automated cell counter (Biorad). No statistical differences were found between the proliferation rates of the two cell lines using multiple t-tests. $P<0.05$.

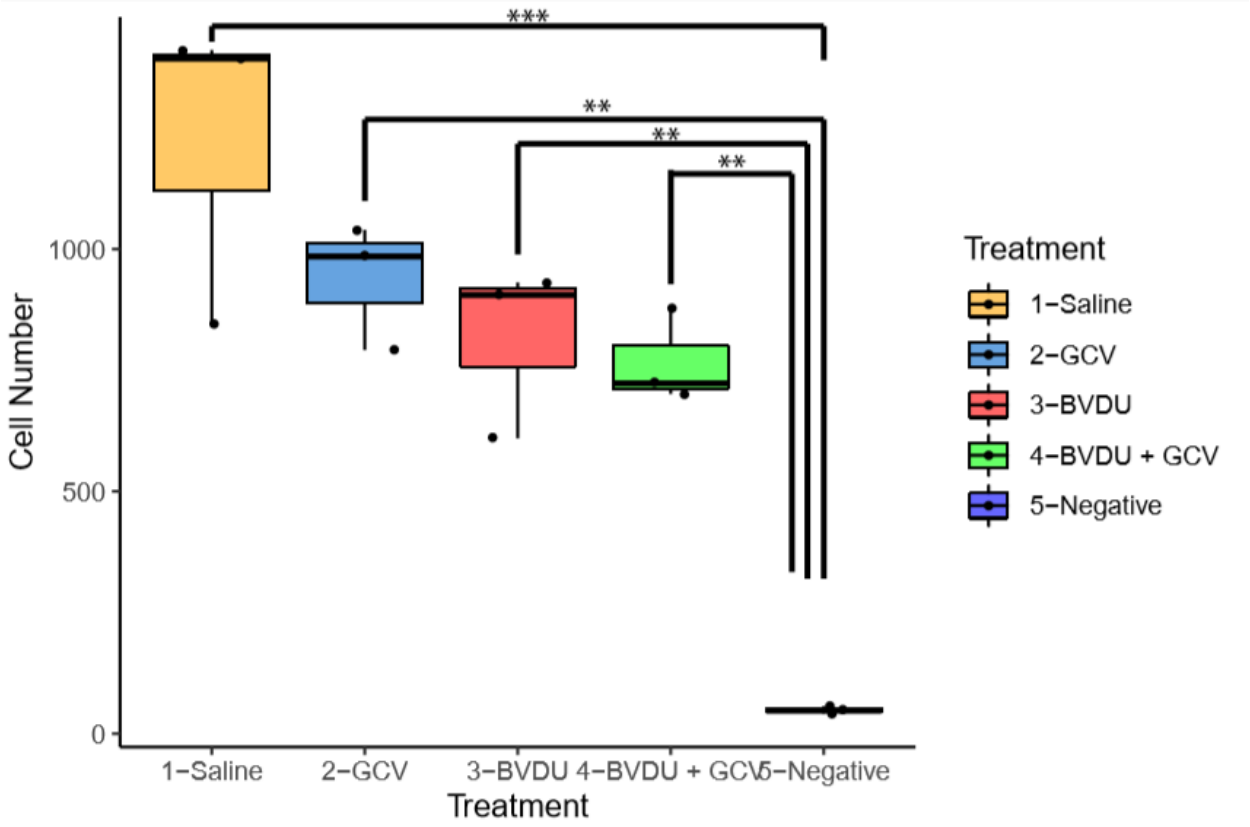

Supplemental Figure 3. Ablation efficiency of GCV and BVDU on transplanted HSV-TK+ NPCs in a rat model with Alu element quantity normalized to harvested cord weight. HSV-TK expressing NPCs were transplanted into a rat model of spinal cord injury, and tail vein injections of 10 mg/kg of BVDU (n=3), 10 mg/kg of GCV (n=3), or 5 mg/kg of both BVDU and GCV (n=3) were given everyday for 4 days. Following the conclusion of injections, 16 days post-transplant, cell survival was assessed by quantifying Alu element copy number from gDNA extracted from harvested spinal cord sections. Alu element quantity was then normalized to total cord weight. None of the treatment groups showed significant differences in cell survival. Data was standardized to gDNA concentration per 1 million rat cells.
